# Gallic Acid Derivatives Propyl Gallate and Epigallocatechin Gallate Reduce rRNA Transcription via Induction of KDM2A Activation

**DOI:** 10.3390/biom12010030

**Published:** 2021-12-25

**Authors:** Yuji Tanaka, Makoto Tsuneoka

**Affiliations:** Laboratory of Molecular and Cellular Biology, Faculty of Pharmacy, Takasaki University of Health and Welfare, Takasaki 370-0033, Japan; tsuneoka@takasaki-u.ac.jp

**Keywords:** rRNA transcription, breast cancer, KDM2A, gallic acid, propyl gallate, epigallocatechin gallate, ROS, histone demethylase

## Abstract

We previously reported that lysine-demethylase 2A (KDM2A), a Jumonji-C histone demethylase, is activated by gallic acid to reduce H3K36me2 levels in the rRNA gene promoter and consequently inhibit rRNA transcription and cell proliferation in the breast cancer cell line MCF-7. Gallic acid activates AMP-activated protein kinase (AMPK) and increases reactive oxygen species (ROS) production to activate KDM2A. Esters of gallic acid, propyl gallate (PG) and epigallocatechin gallate (EGCG), and other chemicals, reduce cancer cell proliferation. However, whether these compounds activate KDM2A has yet to be tested. In this study, we found that PG and EGCG decreased rRNA transcription and cell proliferation through KDM2A in MCF-7 cells. The activation of both AMPK and ROS production by PG or EGCG was required to activate KDM2A. Of note, while the elevation of ROS production by PG or EGCG was limited in time, it was sufficient to activate KDM2A. Importantly, the inhibition of rRNA transcription and cell proliferation by gallic acid, PG, or EGCG was specifically observed in MCF-7 cells, whereas it was not observed in non-tumorigenic MCF10A cells. Altogether, these results suggest that the derivatization of gallic acid may be used to obtain new compounds with anti-cancer activity.

## 1. Introduction

Ribosomes are essential for protein synthesis; in fact, a number of ribosomes affect multiple cellular events [1,2]. Ribosome biogenesis consumes cellular resources, such as materials and energy, which are produced through nutrient metabolism [3,4]. Ribosome RNA (rRNA) transcription is an important step in ribosome construction and is a major factor controlling the number of ribosomes. The rate of rRNA transcription affects various biological events, including cancer cell proliferation [5,6]. The number and size of nucleoli, the sites of ribosome biogenesis, increase with the number of cancer cells; thus, the inhibition of rRNA transcription has been proposed as a therapeutic target to eliminate cancer cells [7].

The chemical modification of histones controls transcription [4,8]. For instance, lysine-specific histone demethylases (KDMs) are known to remove methyl groups from specific residues of histones to control the chromatin structure [9,10,11]. Of note, there are two classes of KDMs: flavin adenine dinucleotide (FAD)-dependent amine oxidases, and Fe(II) and α-ketoglutarate (αKG)-dependent hydroxylases with the Jumonji-C (JmjC) domain. Defects in the enzymatic activity of KDMs are implicated in some diseases, such as developmental disorders and cancers. We previously reported that lysine-demethylase 2A (KDM2A), a JmjC-type KDM, decreases the levels of di-methylated lysine 36 in histone H3 (H3K36me2) but not the tri-methylated form (H3K36me3) in the rRNA gene promoter. KDM2A activity decreased rRNA transcription and the proliferation of breast cancer cells, such as MCF-7 cells, in response to glucose starvation, the anti-diabetic drug metformin, and the food-additive gallic acid [3,4,5-trihydroxybenzoic acid] [6,12,13,14,15]. Under these conditions, the AMP-activated protein kinase (AMPK) was activated, which was required to activate KDM2A [6,14,15]. In MCF-7 cells treated with metformin for 4 h, the intracellular levels of succinate decreased in addition to AMPK activation, both of which are required for the KDM2A activation [14]. On the other hand, although treatment with gallic acid did not lead to a decrease in the intracellular levels of succinate, it caused an elevation in the levels of reactive oxygen species (ROS) in addition to AMPK activation, both of which are required for KDM2A activation [15]. These results suggest that changes in multiple intracellular conditions, including the metabolic and redox statuses, converge to activate KDM2A and control rRNA transcription.

In a previous study, we showed that propyl gallate [propyl 3,4,5-trihydroxybenzoate] (PG) also decreases the proliferation of MCF-7 cells [15]. PG, an ester formed by the condensation of gallic acid and propanol [16], is not a natural compound; it is produced via chemical synthesis. PG is used in the food, cosmetic, and pharmaceutical industries [17]. As a food additive, PG protects fats, oils, and fat-containing foods from rancidity. Importantly, PG was also reported to exhibit anti-tumor properties in HeLa cells and hepatocellular carcinoma cells [18,19]. Additionally, epigallocatechin gallate (EGCG) has been reported to inhibit the proliferation of many types of cancer cells, including colorectal cancer, cervical cancer, and breast cancer cell lines. EGCG is an ester of epigallocatechin and gallic acid and a type of catechin. Importantly, this natural compound is a major active compound of green tea with proven beneficial effects on health, including antioxidant activity, anti-inflammatory activity, and the inhibition of the PI3K/AKT pathway [20,21,22]. It is plausible to consider that in both compounds, ester bonds are cleaved by intracellular esterases to release gallic acid, and are ultimately responsible for the above-mentioned effects. However, it is not clear whether PG and EGCG can activate KDM2A and whether there are any differences between these gallic acid derivatives and gallic acid itself.

To fill this gap in knowledge, in this study, we evaluated the impact of PG and EGCG on the activity of KDM2A in MCF-7 cells. We found that PG and EGCG decreased rRNA transcription through KDM2A in MCF-7 cells. PG and EGCG activated AMPK, and elevated ROS levels, both required for the activation of KDM2A. However, the dynamics of the ROS elevation by PG/EGCG and gallic acid were different. Importantly, we also show that in non-tumor breast epithelium MCF10A cells, PG, EGCG, and gallic acid did not reduce rRNA transcription, activate AMPK, increase ROS production, or impact cell proliferation. Overall, our results show that the gallic acid derivatives PG and EGCG activate KDM2A, consequently suggesting that the esterification of gallic acid does not impact its anti-cancer activity to a major extent.

## 2. Materials and Methods

### 2.1. Chemicals

Gallic acid (Nacalai Tesque INC., Kyoto, Japan; #16520-42), n-propyl gallate (Nacalai Tesque; #29303-92), and EGCG (FUJIFILM Wako Pure Chemical Corporation, Osaka, Japan; #059-05411) were purchased and dissolved in ethanol at 0.5 M concentration (stock solutions). Compound C (IN Solution™ AMPK inhibitor; Merck, Darmstadt, Germany; #171261), N-acetyl L-cysteine (NAC, Nacalai Tesque; #11568-92), glutathione (GSH, Nacalai Tesque; #08786-61), and dimethyl succinate (DMS) (Tokyo Chemical Industry Co., Ltd., Tokyo, Japan.; #S0104) were also purchased and used in this study.

### 2.2. Cell Culture

MCF-7, a human breast adenocarcinoma cell line, was cultured in an RPMI 1640 medium (Nacalai Tesque; #30264-85) supplemented with 10% fetal calf serum (FCS) and 1% penicillin-streptomycin mixed solution (Nacalai Tesque; #09367-34). MCF10A, a human non-tumorigenic epithelial cell line, was cultured in a DMEM/Ham’s F-12 medium with l-Glutamine, sodium pyruvate, and HEPES without phenol red (Nacalai Tesque; #05177-15) supplemented with 5% FCS, 10 μg/mL insulin, 0.5 μg/mL hydrocortisone, 0.1 μg/mL cholera toxin (Sigma Aldrich, St. Louis, MO, USA; #C8052), 20 ng/mL epidermal growth factor, and 1% penicillin-streptomycin. These cells were cultured at 37 °C under humidified 5% CO_2_ conditions.

### 2.3. Evaluation of Cell Proliferation

A CyQUANT^®^ Direct Cell Proliferation Assay kit (Thermo Fisher Scientific, Waltham, MA, USA; #C35011) was used for the evaluation of cell proliferation, as described previously [15]. In brief, 2000 cells were plated in 96-well CELLSTAR microplates (Greiner Bio-One Co., Frickenhausen, Germany; #675090) and treated with compounds, as indicated in the figures. After 2 days of incubation, the cell-permeant DNA-binding dye and background suppressor supplied in the kit were added, and the cells were incubated for 1 h. The signals from the cells were detected using an ARVO MX plate reader (PerkinElmer Inc., Waltham, MA, USA), or a SPARK plate reader (Tecan Inc., Zurich, Switzerland) using standard fluorescein isothiocyanate filter sets. The experiments were performed in triplicates. The values were averaged, and the results are reported as the fold-change relative to the control condition.

### 2.4. Gene Knockdown

Cells were transfected with siRNAs using the Lipofectamine RNAiMAX reagent (Thermo Fisher Scientific; #13778150) according to the manufacturer’s instructions. The siRNA sequence for KDM2A was 5′-GAACCCGAAGAAGAAAGGAUUCGUU-3′, as described previously [12]. The Stealth™ RNAi Negative Control Medium GC Duplex (Thermo Fisher Scientific; #12935300) was also used as control siRNA.

### 2.5. Total RNA Extraction and Real-Time Quantitative PCR

Total RNA was isolated by using the NucleoSpin RNA II kit (Takara Bio Inc., Shiga, Japan; #U0955C), and cDNA was synthesized from the total RNA using the Superscript III First-strand Synthesis System (Thermo Fisher Scientific; #18080051) with random hexamers. The products were subjected to qRT-PCR using the KAPA SYBR FAST qPCR Master Mix reagent (Nippon Genetics Co. Ltd., Tokyo, Japan; #KR0389) and a CFX Connect real-time PCR detection system (Bio-Rad Laboratories, Inc., Berkeley, CA, USA). The expression levels of the target genes were normalized with the level of β-actin mRNA and are reported as the ratio to the control conditions. The pre-rRNA level was measured to evaluate the levels of rRNA transcription. The primers used to amplify the pre-rRNA followed those used in a previous study [6]: 5′-GCTGACACGCTGTCCTCTG-3′ and 5′-TCGGACGCGCGAGAGAAC-3′. For KDM2A mRNA, the primers used were 5′-TCCCCACACACATTTTGACATC-3′ and 5′-GGGGTGGCTTGAGAGATCCT-3′. For β-actin mRNA, the primers used were 5′-CGTCTTCCCCTCCATCGT-3′ and 5′-GAAGGTGTGGTGCCAGATTT-3′.

### 2.6. Antibodies

An anti-dimethylated histone H3 lys36 antibody (MAB Institute Inc., Nagano, Japan; #MABI0332-100), anti-trimethylated histone H3 lys36 antibody (MAB Institute Inc.; #MABI0333-100), anti-histone H3 antibody (Takara Bio Inc.; #MA301B), and a control normal rabbit IgG antibody (Cell Signaling Technology (CST), Danvers, MA, USA.; #2729S) were used in this study for chromatin immunoprecipitation (ChIP). An anti-KDM2A antibody, described previously [12], was also used in this study. The anti-phosphorylated AMPKα (Thr-172) (CST; #2535), anti-AMPKα (CST; #5831), and anti-β-actin (Sigma; #A1978) antibodies were used for immunoblotting.

### 2.7. ChIP Assay

A ChIP assay using Dynabeads^®^ protein G (Thermo Fisher Scientific; #10003D) was performed, as described previously [15]. The DNA fragments collected by ChIP assay were quantified by qRT-PCR. The primers used for the detection of the rRNA gene promoter (+1 to +155 from the transcriptional start site) were the same primer set used to detect pre-rRNA. To evaluate specific binding, the values from the qRT-PCR were divided by those from the input amplification (% of input), and the values obtained in the control antibody (normal rabbit IgG) were subtracted. Additionally, for histone modifications, the values were normalized to those obtained by using the normal histone H3 antibody (% of specific bound/input normalized to H3). The experiments were repeated more than three times. The ratios and standard deviations calculated from these values are reported.

### 2.8. Immunoblotting

After treatment, cells were harvested to extract protein with an SDS-PAGE sampling buffer (4% SDS solution containing 100 mM Tris, pH 6.8, 50 mM DTT, and 20% glycerol). The same protein concentration of each extract was then subjected to SDS-PAGE, and the resolved proteins were transferred onto a PVDF membrane (Millipore, Burlington, MA, USA; #IPVH00010). After incubation with specific antibodies according to the manufacturer’s instructions, protein bands were detected using the Immobilon Western system (Millipore; #WBKLS0100), as described previously [12,15]. The intensities of the bands were measured by Image J software. The effects of the treatments are expressed as the ratio to those without treatment.

### 2.9. DCFDA Assay

The DCFDA/H2DCFDA-Cellular ROS Assay Kit (Abcam, Cambridge, UK; #ab113851) was used to measure the intracellular ROS levels, as previously described [15]. Briefly, 20,000 cells were plated in a 96-well microplate. After overnight culture, these cells were washed with the 1x buffer solution supplied in the kit, and treated with 20 μM DCFDA solution in 1x buffer for 45 min. After washing twice with PBS, the cells were placed in the culture medium containing compounds, and the DCF signals were measured at various times using an ARVO MX plate reader (PerkinElmer Inc.) or a SPARK plate reader (Tecan Inc.), with an excitation/emission wavelength of 485/535 nm. The values obtained from the blank condition (no cells) were subtracted from the DCF signal values.

### 2.10. Statistical Analysis

The *p*-values in all figures were calculated using one-way ANOVA with Tukey’s HSD in the EZR graphical interface for R [23].

## 3. Results

### 3.1. PG and EGCG Induce the KDM2A-Dependent Reduction of rRNA Transcription in MCF-7 Cells

Previously, we demonstrated that the treatment of MCF-7 cells with 50 μM gallic acid induced the activity of KDM2A, leading to the reduction of H3K36me2 in the rRNA gene promoter and consequently of rRNA transcription [15]. Although both PG and EGCG have gallic acid residues (Figure S1), it is still unknown whether these compounds also modulate the activity of KDM2A and control the transcription of rRNA. To address this gap in knowledge, here, we investigated the effect of PG and EGCG in the KDM2A-dependent regulation of rRNA transcription in comparison to that of gallic acid.

**Figure 1:**
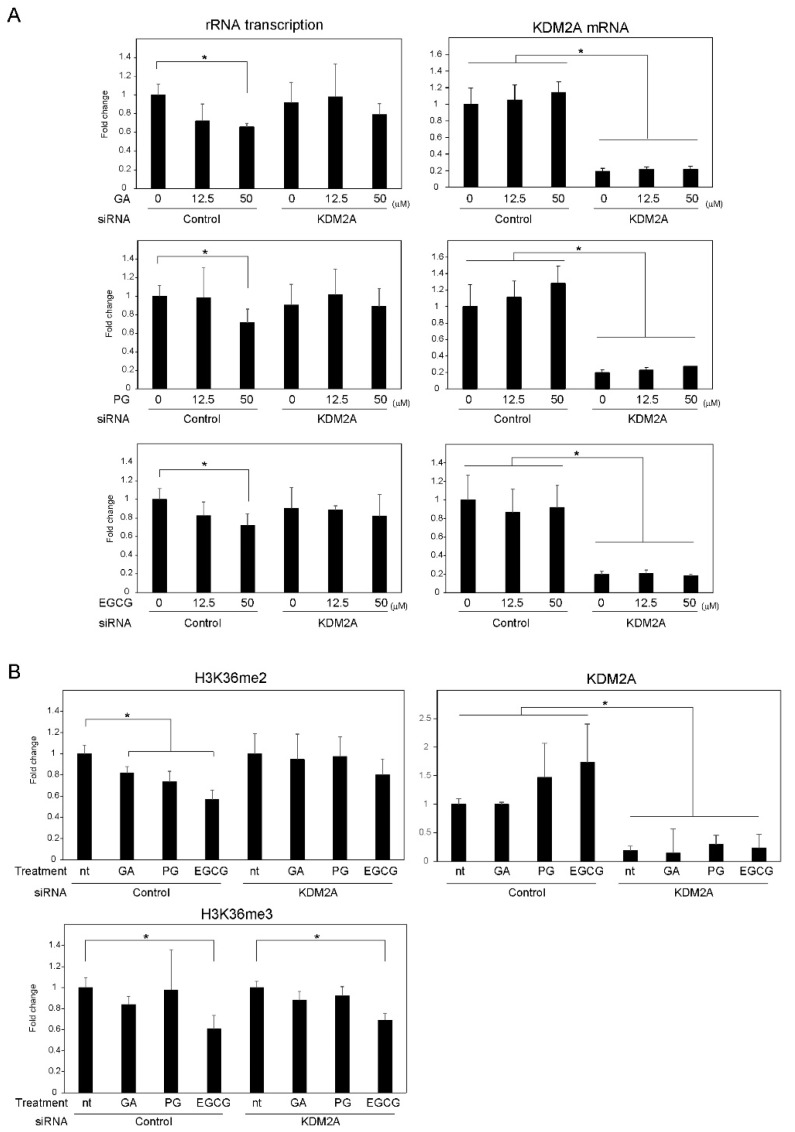
Propyl gallate (PG) and epigallocatechin gallate (EGCG) decrease rRNA transcription and H3K36me2 levels in the rRNA gene promoter through KDM2A. (**A**) MCF-7 cells transfected with control siRNA or KDM2A siRNA were treated with the indicated concentrations of gallic acid (GA), PG or EGCG for 4 h. Total RNA was extracted, and the levels of rRNA transcripts (pre-rRNA) (left panel) and KDM2A mRNA (right panel) were determined using quantitative real-time PCRs (qRT-PCR). The results are shown as the fold change in relation to cells treated with control siRNA in the absence of compounds. (**B**) MCF-7 cells transfected with control siRNA or KDM2A siRNA were treated with 50 μM GA, PG or EGCG for 4 h. The levels of H3K36me2, H3K36me3, and KDM2A in the rRNA gene promoter were analyzed by ChIP assay. The results are shown the fold change in relation to cells treated with control siRNA in the absence of compounds. All experiments were performed more than three times, and the mean values with standard deviations are shown. * *p* < 0.05.

First, MCF-7 cells were treated with gallic acid, PG, or EGCG for 4 h, at concentrations of 0, 12.5 and 50 μM, and the levels of rRNA transcription were detected (Figure 1A). Treatment with gallic acid and EGCG starting from the concentration of 12.5 μM decreased rRNA transcription in a dose-dependent manner; KDM2A knockdown alleviated this decrease (Figure 1A, GA and EGCG). Additionally, treatment with 50 μM PG also decreased rRNA transcription; again, KDM2A knockdown alleviated this decrease (Figure 1A, PG). Of note, the levels of *KDM2A* mRNA were not significantly altered by PG or EGCG. These results suggest that PG and EGCG also have the ability to reduce rRNA transcription through KDM2A.

Treatment of MCF-7 cells with gallic acid, PG, or EGCG at a concentration of 50 μM for 4 h also decreased the levels of histone H3K36me2, a direct substrate of KDM2A, in the rRNA gene promoter (Figure 1B, control). Such a decrease by the three compounds was alleviated after KDM2A knockdown (Figure 1B). Of note, the levels of histone H3K36me3 in the rRNA gene promoter in gallic acid-, PG-, and EGCG-treated cells were not significantly changed by KDM2A knockdown (Figure 1B). Altogether, these results demonstrate that PG and EGCG, in addition to gallic acid, induce KDM2A demethylase activity in the rRNA gene promoter and decrease rRNA transcription.

The activity of JmjC enzymes, including KDM2A, requires α-ketoglutarate (α-KG), which is converted into succinate. Dimethyl succinate (DMS), a cell-permeable succinate, inhibited the demethylation activity of KDM2A [12]. In fact, previously, the reduction of rRNA transcription and of the levels of H3K36me2 in the rRNA gene promoter by gallic acid was demonstrated to be inhibited by DMS treatment [15]. Here, to confirm that the demethylase activity is involved in the reduction of rRNA transcription by PG and EGCG, DMS was added to the medium, together with these compounds. The effect of both PG and EGCG on rRNA transcription (Figure S2A), and the levels of H3K36me2 in the rRNA gene promoter (Figure S2B) was also alleviated by the addition of 5 mM DMS. Therefore, these results suggest that the repression of rRNA transcription by PG, EGCG, and gallic acid occurs due to the demethylase activity of KDM2A.

### 3.2. PG- and EGCG-Mediated Activation of AMPK Is Required for the Reduction of rRNA Transcription in MCF-7 Cells

**Figure 2:**
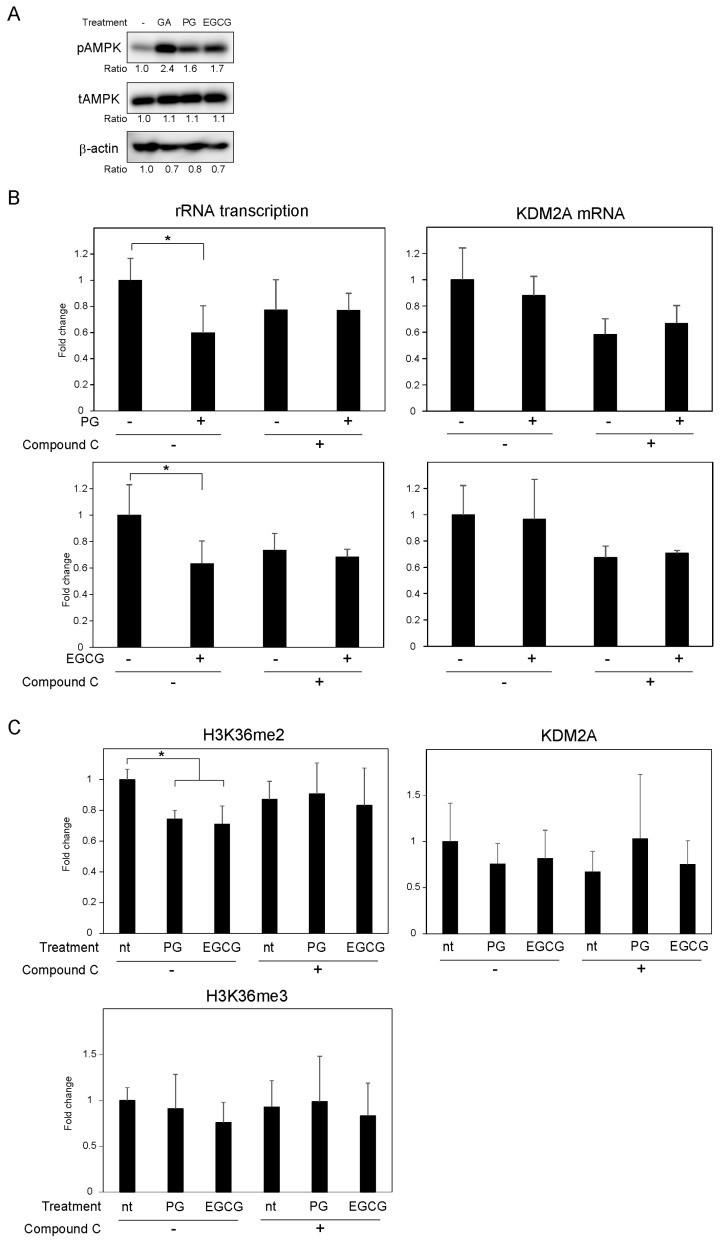
PG- and EGCG-mediated activation of AMPK is required for the decrease in the levels of histone H3K36me2 in the rRNA gene promoter and consequent rRNA transcription. (**A**) MCF-7 cells were treated with 50 μM GA, PG, or EGCG for 4 h. The cells were then lysed, and the levels of phosphorylated-AMPKα (Thr172) (pAMPK), total AMPKα (tAMPK), and β-actin were analyzed via immunoblotting. (**B**) MCF-7 cells were treated with (+) or without (-) 50 μM PG or EGCG in the presence (+) or absence (-) of 10 μM compound C, an AMPK inhibitor, for 4 h. Total RNA was extracted, and the levels of rRNA transcripts (pre-rRNA) (left panel) and KDM2A mRNA (right panel) were determined using qRT-PCR. The results are shown as the fold change in relation to cells in the absence of compounds. (**C**) MCF-7 cells were treated with or without 50 μM PG or EGCG in the presence (+) or absence (-) of 10 μM compound C for 4 h. The levels of H3K36me2, H3K36me3, and KDM2A in the rRNA gene promoter were analyzed by ChIP assay. The results are shown as the fold change in relation to cells in the absence of compounds. The experiments in (**B**,**C**) were performed more than three times, and the mean values with standard deviations are shown. * *p* < 0.05.

Previously, the gallic acid-mediated activation of AMPK was required for the activation of KDM2A and the reduction of rRNA transcription [15]. Therefore, next, we investigated whether PG and EGCG also activated AMPK in MCF-7 cells. When MCF-7 cells were treated with gallic acid, PG, and EGCG at 50 μM for 4 h, the levels of Thr172 phosphorylated AMPKα (an AMPK activation marker) were increased (Figure 2A). Of note, the levels of total AMPK and β-actin were comparable after these treatments, suggesting that PG and EGCG are indeed able to activate AMPK.

To test whether the activation of AMPK was required for the reduction of rRNA transcription through KDM2A, MCF-7 cells were treated with compound C, an inhibitor of AMPK, in the presence of PG or EGCG. Treatment with compound C prevented the reduction of rRNA transcription by 50 μM PG or EGCG (Figure 2B). The reduction in the H3K36me2 levels in the rRNA gene promoter by 50 μM PG or EGCG for 4 h was inhibited by treatment with compound C (Figure 2C). Altogether, these results suggest that the PG- or EGCG-mediated KDM2A-dependent reduction of rRNA transcription requires the activation of AMPK.

### 3.3. PG and EGCG Increase ROS Levels for Reduction in rRNA Transcription in MCF-7 Cells

**Figure 3:**
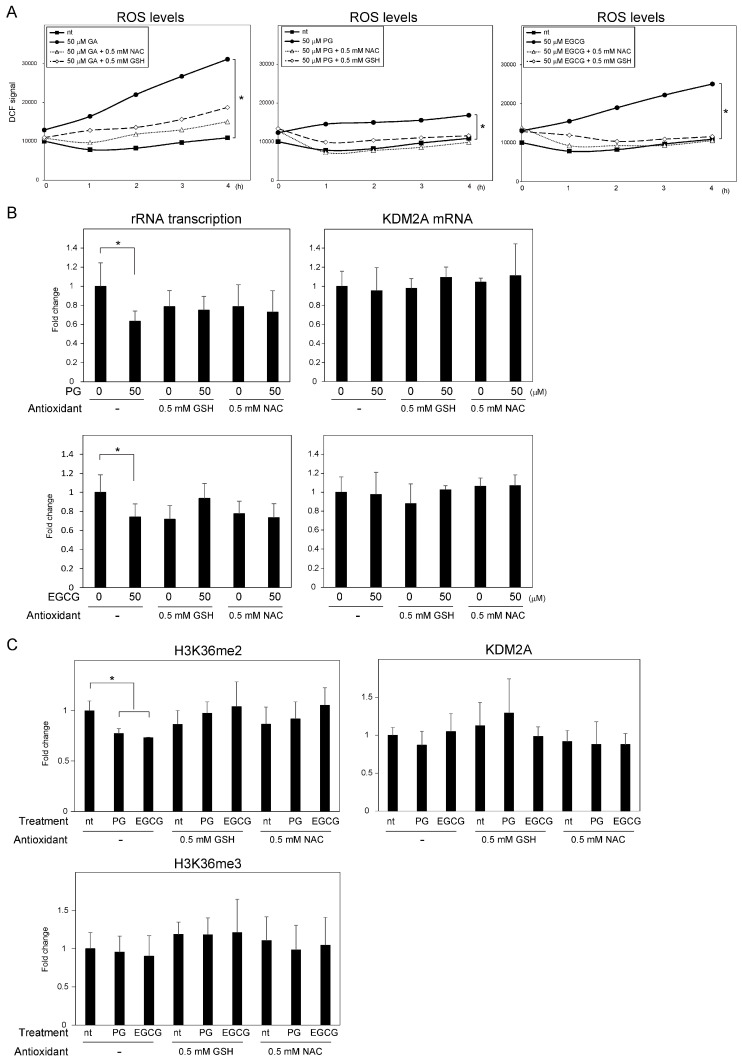
PG- and EGCG-induced elevation of ROS levels is required for the decrease in the levels of histone H3K36me2 in the rRNA gene promoter and consequent rRNA transcription. (**A**) MCF-7 cells were treated with 50 μM GA, PG, or EGCG with or without glutathione (GSH) or N-acetyl-L-cysteine (NAC), and cultured in the presence of DCFDA, a cell-permeable ROS probe. Fluorescence was measured at the indicated time-point. The signal intensity was normalized to that of blank condition (no cells) without treatments; the mean values (n = 3) are shown. (**B**) MCF-7 cells were treated with or without 50 μM PG or EGCG, in the presence or absence of 0.5 mM GSH or 0.5 mM NAC, for 4 h. Total RNA was extracted, and the levels of rRNA transcripts (pre-rRNA) (left panel) and KDM2A mRNA (right panel) were determined using qRT-PCR. The results are shown as the fold change in relation to cells in the absence of compounds. (**C**) MCF-7 cells were treated with or without 50 μM PG or EGCG, in the presence or absence of 0.5 mM GSH or 0.5 mM NAC, for 4 h. The levels of H3K36me2, H3K36me3, and KDM2A in the rRNA gene promoter were analyzed via ChIP. The results are shown as the fold change in relation to cells in the absence of compounds. The experiments in (**B**,**C**) were performed more than three times, and the mean values with standard deviations are shown. * *p* < 0.05.

We previously reported that the elevation of ROS production by gallic acid is required for the reduction of rRNA transcription through KDM2A [15]. To disclose whether the same is true for PG or EGCG, MCF-7 cells were treated with 50 μM GA, PG, or EGCG, and the ROS levels were measured using the DCFDA probe (Figure 3A). The four-hour-treatment with either EGCG or PG-elevated ROS production, and the co-incubation with N-acetyl-L-cysteine (NAC) or glutathione (GSH) decreased such an elevation, as previously reported for gallic acid (Figure 3A) [15]. Of note, the levels of ROS produced in response to PG, and to a lesser extent by EGCG, were lower than those produced by gallic acid, which were elevated. Additionally, after 24 h of treatment, the levels of ROS after 50 μM gallic acid treatment were as elevated as those determined at 4 h (Figure S3). On the other hand, after 24 h of treatment with 50 μM EGCG or PG, the levels of ROS were no longer significantly increased (Figure S3). Altogether, these results suggest that PG and EGCG cause an increase in ROS levels, but contrary to that observed after gallic acid treatment, such an increase was of a lower magnitude and transient. To investigate whether the transient elevation of ROS caused by PG and EGCG was required for the reduction of rRNA transcription, the antioxidants GSH and NAC were added to the medium in the presence of 50 μM PG or EGCG. The reduction of rRNA transcription was not detected when PG or EGCG were co-incubated with GSH or NAC for 4 h (Figure 3B). Additionally, the decrease in the H3K36me2 levels in the rRNA gene promoter after treatment with PG or EGCG was not detected in the MCF-7 cells treated with these antioxidants (Figure 3C). Therefore, these results suggest that the PG- and EGCG-induced elevation of the ROS levels in MCF-7 cells is required for the KDM2A-mediated decrease in H3K36me2 levels in the rRNA gene promoter, and the consequent downregulation of rRNA transcription.

### 3.4. PG and EGCG Reduce the Proliferation of MCF-7 Cells but Not of MCF10A Cells

**Figure 4:**
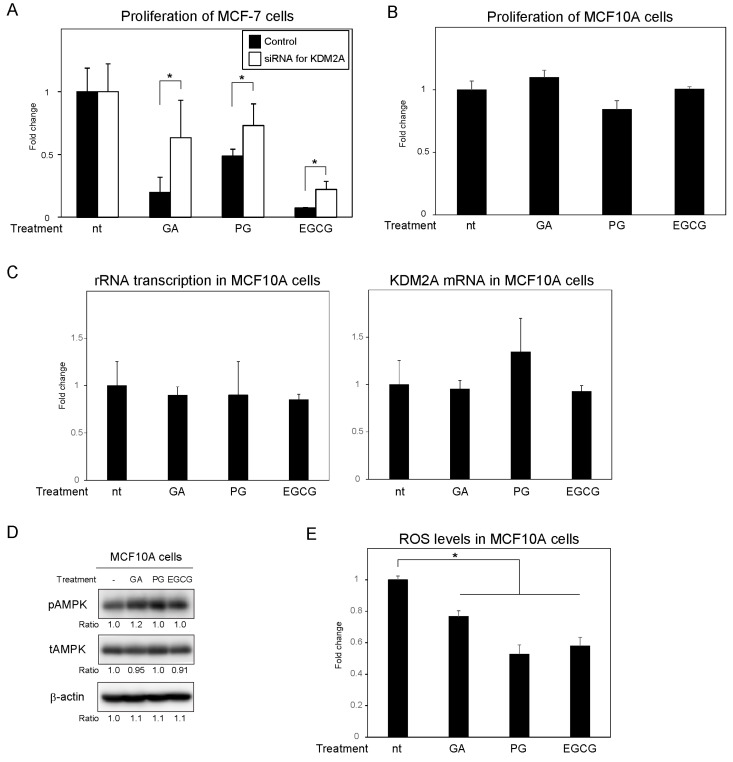
Impact of PG and EGCG on the proliferation of MCF-7 and MCF10A cells. (**A**) MCF-7 cells transfected with control siRNA or KDM2A siRNA were treated with 50 μM GA, PG or EGCG for 2 days. Cell proliferation was analyzed using the CyQUANT^®^ direct cell proliferation assay. The results are shown as the fold change in relation to cells treated with siRNA in the absence of compounds. (**B**) MCF10A cells were treated with 50 μM GA, PG, or EGCG for 2 days. Cell proliferation was analyzed, as in (**A**). The results are shown as the fold change in relation to cells in the absence of compounds. (**C**) MCF10A cells were treated with 50 μM GA, PG, or EGCG for 4 h. Total RNA was extracted, and the levels of rRNA transcripts (pre-rRNA) (left panel) and KDM2A mRNA (right panel) were determined using qRT-PCR. The results are shown as the fold change in relation to cells in the absence of compounds. (**D**) MCF10A cells were treated with 50 μM GA, PG, or EGCG for 4 h. The cells were then lysed and the levels of phosphorylated-AMPKα (Thr172) (pAMPK), total AMPKα (tAMPK), and β-actin were analyzed via immunoblotting. (**E**) MCF10A cells were treated with 50 μM GA, PG, or EGCG and cultured for 4 h in the presence of DCFDA, a cell-permeable ROS probe. Fluorescence was measured and the signal intensity was normalized to that of blank condition (no cells) without treatments. The results are presented as the fold change from cells without treatment. All experiments were performed more than three times, and the mean values with standard deviations are shown. * *p* < 0.05.

Next, we investigated whether similar to gallic acid, PG and EGCG reduce the proliferation of MCF-7 cells through KDM2A. After MCF-7 cells were treated with gallic acid, PG, or EGCG at a concentration of 50 μM for 2 days, the cell numbers were determined using the CyQUANT direct cell proliferation assay. PG, EGCG, and gallic acid all reduced cell proliferation, and KDM2A knockdown partially abrogated this effect (Figure 4A), suggesting that gallic acid derivatives have the ability to reduce cell proliferation in a KDM2A-dependent manner. Of note, the reduction in cell proliferation by PG was weaker than that by gallic acid and EGCG (Figure 4A). Altogether, these results suggest that although gallic acid, PG, and EGCG are all able to reduce the proliferation of MCF-7 cells in a KDM2A-dependent manner, they do it to a different extent. Previously, it was shown that treatment with 50 μM gallic acid did not affect the proliferation of non-tumorigenic MCF10A cells [15]. Therefore, next, we also evaluated the effects of PG and EGCG on the proliferation of MCF10A cells. Reproducing our previous results, treatment with gallic acid at a concentration of 50 μM did not decrease the proliferation of MCF10A cells (Figure 4B); importantly, the same was true for 50 μM PG or EGCG. Additionally, treatment with 50 μM PG or EGCG neither decreased rRNA transcription (Figure 4C), induced AMPK activation (Figure 4D), or elevated the level of ROS (Figure 4E) in MCF10A cells. In fact, treatment with PG, EGCG, or gallic acid did not elevate the ROS levels in MCF10A cells (Figure 4E); of note, the reduction in the ROS levels was higher after both PG and EGCG treatment than that observed after gallic acid treatment. Altogether, these results suggest that PG and EGCG have a selective effect on cancer cells.

## 4. Discussion

We previously demonstrated that gallic acid reduces rRNA transcription and impacts the proliferation of MCF-7 breast cancer cells in a KDM2A-dependent manner [15]. In this study, we investigated the effects of two compounds containing gallic acid in their structure, PG and EGCG on rRNA transcription and cell proliferation in the same cancer cell line. Of note, PG was previously shown by us to decrease the proliferation of MCF-7 cells [15]. PG and EGCG have also been widely reported to exhibit anti-proliferative activity [20,22]. However, it has not yet been investigated whether PG and EGCG activate KDM2A to reduce rRNA transcription in cancer cells. Here, we show that treatment with PG and EGCG at a concentration of 50 μM reduced the H3K36me2 levels in the rRNA gene promoter, and consequently rRNA transcription in a KDM2A-dependent manner (Figure 1). Therefore, these results suggest that the esters formed by the condensation of gallic acid and propanol or epigallocatechin reduce rRNA transcription in a KDM2A dependent manner. We also showed that PG and EGCG promote the activation of AMPK, which is required for the reduction of the H3K36me2 levels in the rRNA gene promoter and of rRNA transcription (Figure 2). Elevated ROS levels after PG and EGCG treatment were also observed in MCF-7 cells. However, the treatment of MCF-7 cells with EGCG increased ROS to levels similar to those observed after treatment with gallic acid, and PG treatment only weakly increased ROS production. Additionally, the elevation of the ROS levels caused by PG and EGCG was mostly lost 24 h after treatment, whereas the increase in ROS levels was maintained in MCF-7 cells treated with gallic acid for 24 h (Figure S3). That said, the elevation of ROS was required for the KDM2A-dependent reduction of rRNA transcription, because antioxidants inhibited the effects of PG, EGCG, and gallic acid in MCF-7 cells (Figure 3). Therefore, these results suggest that the transient elevation of ROS levels is sufficient to induce a KDM2A-dependent reduction of rRNA transcription. The anti-proliferative activity of both PG and EGCG in MCF-7 cells was also evident. However, the decrease in the number of MCF-7 cells by PG was weaker than that caused by gallic acid or EGCG (Figure 4A). On the other hand, the decrease in the levels of H3K36me2 in the rRNA gene promoter and the decrease in rRNA transcription in MCF-7 cells treated with PG and EGCG was comparable to that observed after gallic acid treatment (Figure 1). Therefore, the differences in the anti-proliferative activity of the three compounds are probably dependent on factors other than the control of rRNA transcription. It has been reported that gallic acid down-regulates pathways involved in cancer progression, such as PI3K/Akt [24]. Therefore, we speculate that this signaling pathway may also affect the anti-proliferative activity of gallic acid derivatives. Previously, we also demonstrated that 50 μM gallic acid did not impact the proliferation of non-tumorigenic MCF10A cells [15]. PG and EGCG showed a similar effect in this study (Figure 4). These results suggest that the esterification of gallic acid did not affect its selectivity toward cancer cells. Importantly, we showed that while the gallic acid derivatives promoted the activation of AMPK and increased ROS levels, which ultimately led to the reduction of rRNA transcription in MCF-7 cells in a KDM2A-dependent manner, the same was not observed in non-tumorigenic MCF10A cells (Figure 4). However, the mechanisms underlying these differences between MCF-7 and MCF10A cells remain unclear. Further studies are required to understand the mechanism behind the selectivity of gallic acid and its derivatives toward cancer cells.

Overall, our results suggest that the combination of esterified gallic acid and clinically used anti-cancer drugs may be a good strategy to increase the selectivity of these drugs toward cancer cells. Of note, the anticancer activity of drugs, such as cisplatin and paclitaxel, has been suggested to be enhanced in combination therapy with gallic acid [25,26,27]. Additionally, our results point to PG, already used as a food additive, and EGCG, contained in food sources such as green tea, as potential anticancer agents. In fact, these compounds are easy to obtain and safe. Importantly, further studies may provide more gallic acid derivatives that activate KDM2A and reduce rRNA transcription, and reveal novel anti-cancer agents.

## Data Availability

Not applicable.

## Supplementary Materials

The following are available online at https://www.mdpi.com/article/10.3390/biom12010030/s1, Figure S1: Chemical structures of gallic acid, propyl gallate (PG), and epigallocatechin gallate (EGCG), Figure S2: Dimethyl succinate (DMS) prevents the effects of propyl gallate (PG) and epigallocatechin gallate (EGCG) on rRNA transcription in MCF-7 cells, Figure S3: Reactive oxygen species (ROS) levels in MCF-7 cells treated with GA, PG, and EGCG for 24 h.
