# Supplementary material for: Gallic Acid Derivatives Propyl Gallate and Epigallocatechin Gallate Reduce rRNA Transcription via Induction of KDM2A Activation
Source: Biomolecules. 2021 Dec 25;12(1):30. doi: 10.3390/biom12010030 (PMC8773796; doi:10.3390/biom12010030)
Supplement: Supplementary file 1 [file biomolecules-12-00030-s001.zip › biomolecules-1487357-supplementary.pdf]

biomolecules

MDPI

Supplementary Materials

Gallic Acid Derivatives Propyl Gallate and Epigallocatechin Gallate reduce rRNA Transcription via KDM2A

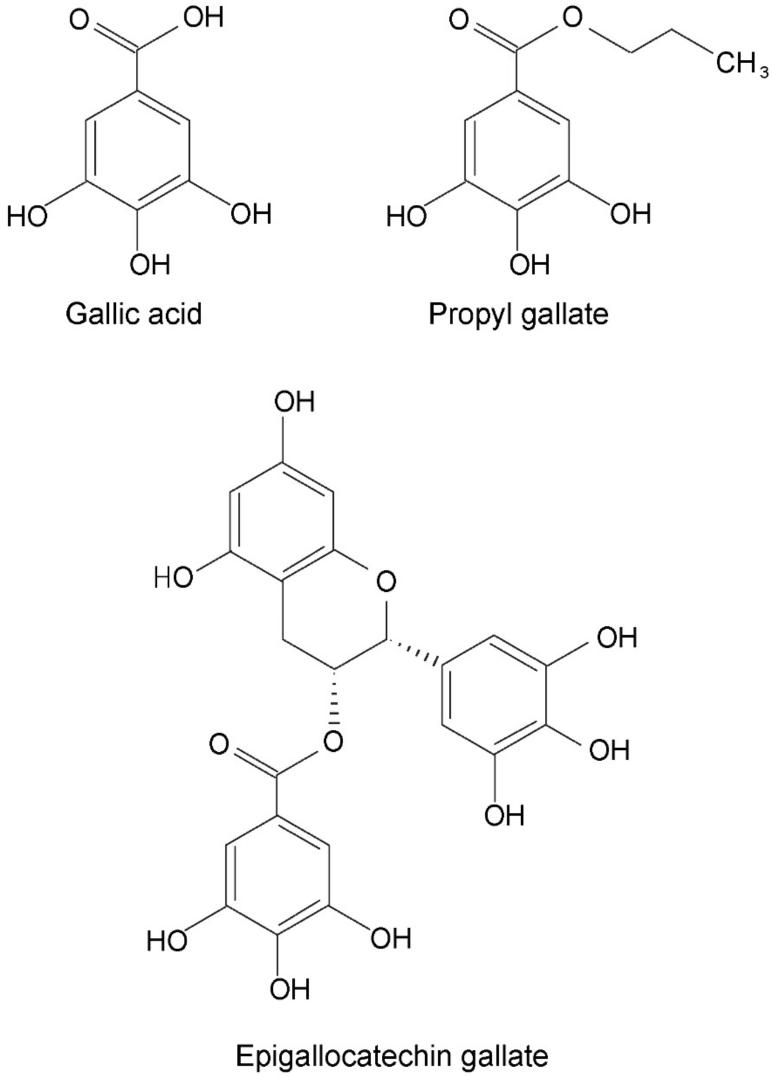

Figure S1. Chemical structures of gallic acid, propyl gallate (PG), and epigallocatechin gallate (EGCG). PG and EGCG contain at least one gallic acid residue.

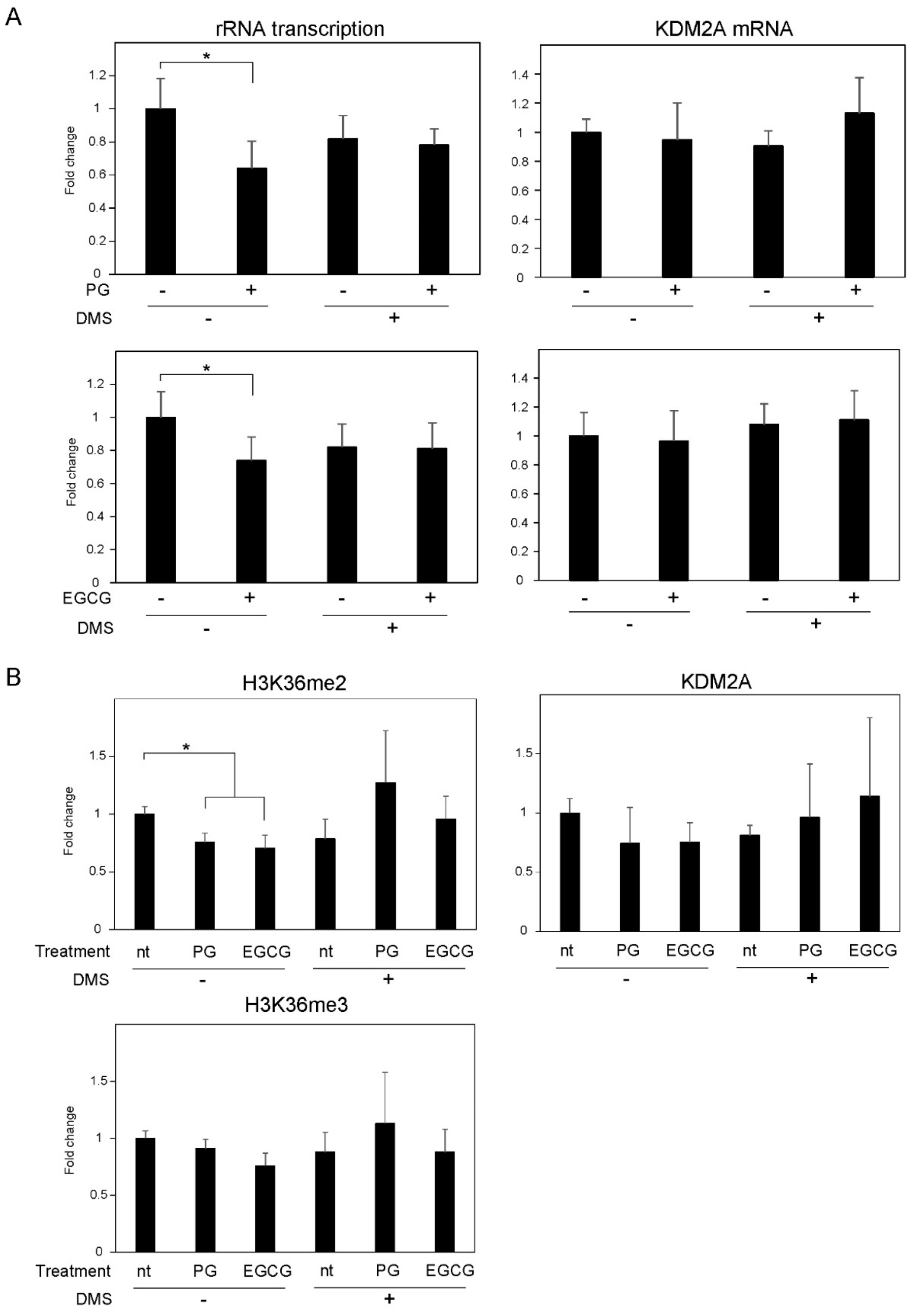

Figure S2. Dimethyl succinate (DMS) prevents the effects of propyl gallate (PG) and epigallocatechin gallate (EGCG) on rRNA transcription in MCF-7 cells. (**A**) MCF-7 cells were treated with (+) or without (-) 50 µM PG (upper panel) or 50 µM EGCG (lower panel) in the presence (+) or absence (-) of 5 mM DMS for 4 h. Total RNA was extracted, and the levels of rRNA transcripts (pre-rRNA) (left panel) and KDM2A mRNA (right panel) were determined using quantitative real-time PCR (qRT-PCR). The results are shown as the fold change in relation to cells in the absence of compounds. (**B**) MCF-7 cells were treated with 50 µM PG or EGCG in the presence (+) or absence (-) of 5 mM DMS for 4 h. The levels of H3K36me2, H3K36me3, and KDM2A in the rRNA gene promoter were analyzed using ChIP. The results are shown as the fold change in relation to cells in the absence of compounds. All experiments were performed more than three times, and the mean values with standard deviations are shown. * $p < 0.05$.

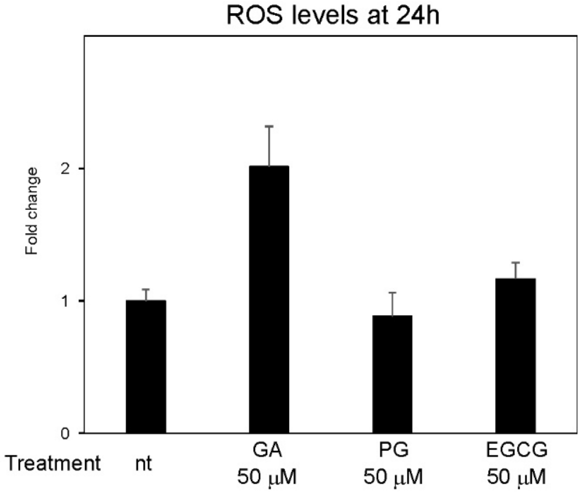

Figure S3. Reactive oxygen species (ROS) levels in MCF-7 cells treated with gallic acid (GA), propyl gallate (PG) and epigallocatechin gallate (EGCG) for 24 h. MCF-7 cells were treated with 50 μM GA, PG, or EGCG and cultured in the presence of DCFDA, a cell-permeable ROS probe, for 24 h. Fluorescence was measured, and the signal intensity was normalized to that of a no cell condition. The results are shown as the fold change to cells without treatments. The mean values ± the standard deviation (n = 3) are shown.
